# Comparison of outcomes between early-stage cervical cancer patients without high-risk factors undergoing adjuvant concurrent chemoradiotherapy and radiotherapy alone after radical surgery

**DOI:** 10.1186/s12885-024-12284-9

**Published:** 2024-04-30

**Authors:** Yuncan Zhou, Weiping Wang, Jia Tang, Ke Hu, Fuquan Zhang

**Affiliations:** Department of Radiation Oncology, Peking Union Medical College Hospital, Chinese Academy of Medical Sciences and Peking Union Medical College, Beijing, China

**Keywords:** Cervical cancer, Deep stromal invasion, Large tumor size, Lymphovascular space involvement, Postoperative concurrent radiochemotherapy, Radiotherapy

## Abstract

**Purpose:**

For patients with early-stage cervical cancer without high-risk factors, there is no consensus regarding the optimal postoperative treatment regimen and whether postoperative concurrent radiochemotherapy (CCRT) is superior to radiotherapy (RT) alone.

**Patients and methods:**

The medical records of patients with stage I-IIA cervical cancer, who underwent radical surgery and postoperative RT or CCRT between June 2012 and December 2017, were retrospectively reviewed. Patients with any high-risk factors, including positive pelvic lymph node(s), positive resection margin(s), and parametrial invasion, were excluded. Patients with large tumors (≥ 4 cm), deep stromal invasion (≥ 1/2), and lymphovascular space involvement were categorized as the intermediate-risk group. Patients without intermediate-risk factors were categorized as the low-risk group.

**Results:**

A total of 403 patients were enrolled and divided into 2 groups according to postoperative treatment: RT alone (*n* = 105); and CCRT (*n* = 298). For risk stratification, patients were also divided into 2 groups: intermediate-risk (*n* = 350); and low-risk (*n* = 53). The median follow-up was 51.7 months. Patients in the intermediate-risk group and those with multiple intermediate-risk factors were more likely to undergo CCRT. For patients who underwent RT alone or CCRT in the intermediate-risk group, 5-year overall survival (OS) rates were 93.4% and 93.8% (*p* = 0.741), and 5-year disease-free survival (DFS) rates were 90.6% and 91.4%, respectively (*p* = 0.733). Similarly, for patients who underwent RT alone or CCRT in the low-risk group, the 5-year OS rates were 100.0% and 93.5% (*p* = 0.241), and 5-year DFS rates were 94.4% and 93.5%, respectively (*p* = 0.736). Adjuvant CCRT or RT were not independent risk factors for either OS or DFS. Patients who underwent CCRT appeared to develop a higher proportion of grade ≥ 3 acute hematological toxicities than those in the RT group (44.0% versus 11.4%, respectively; *p* < 0.001). There was no significant difference in grade ≥ 3 chronic toxicities of the urogenital and gastrointestinal systems between the CCRT and RT groups.

**Conclusion:**

There was no significant difference in 5-year OS and DFS rates between patients with early-stage cervical cancer without high-risk factors undergoing postoperative CCRT versus RT alone. Patients who underwent CCRT appeared to develop a higher proportion of grade ≥ 3 acute hematological toxicities than those who underwent RT alone.

## Background

Cervical cancer is among several gynecological tumors with a high incidence rate. The International Agency for Research on Cancer estimated that there were 604,127 new cases and 341,831 cancer-related deaths due to cervical cancer worldwide in 2020 [1]. Although cytological screening has played an important role in the early diagnosis of cervical cancer, the number of new cases and deaths remains high.

Treatment of cervical cancer is based on clinical and pathological features. Based on clinical practice guidelines from the National Comprehensive Cancer Network (NCCN), the primary treatment of early-stage cervical cancer is either radical surgery or radiotherapy (RT). For patients who have undergone primary surgery, the postoperative adjuvant therapy regimen depends on the International Federation of Gynecology and Obstetrics (FIGO) stage and pathological risk factors of surgical findings [2].

In terms of surgical pathology, positive resection margin(s), parametrial invasion, and positive pelvic lymph node(s) are high-risk factors for cervical cancer, for which postoperative concurrent radiochemotherapy (CCRT) is recommended [2, 3]. For patients who meet the Sedlis criteria, postoperative radiotherapy is the standard treatment regimen, which is recommended by NCCN guideline. However, for patients without any high-risk factors, no consensus has been reached regarding whether postoperative CCRT is superior to RT alone.

Based on earlier studies, deep stromal invasion, large primary tumor size, and lymphovascular space involvement (LVSI) are considered to be intermediate-risk factors [2, 4–7]. Several studies have demonstrated that, compared with postoperative RT alone, CCRT may improve prognosis in patients with intermediate-risk factors, [8, 9] although more recent studies have come to the opposite conclusion. Mahmoud et al. [10] enrolled 869 patients with early-stage cervical cancer with intermediate-risk factors who underwent adjuvant CCRT (*n* = 440) or RT (*n* = 429) after radical surgery. Results revealed that, compared with patients who underwent RT, there was no survival benefit for those who underwent CCRT. Kim et al. [11] and Qin et al. [12] also demonstrated that postoperative CCRT was not superior to RT alone for patients with intermediate-risk factors. Moreover, studies focusing on postoperative adjuvant treatment for patients without any high- or intermediate-risk factors remain limited.

The primary objective of this retrospective study, therefore, was to compare outcomes between patients without high-risk factors who underwent postoperative CCRT versus RT alone after primary radical surgery.

## Methods

### Study population

The present study retrospectively collected data from the medical records of patients diagnosed with cervical cancer between June 2012 and December 2017 at the authors’ institution. Inclusion criteria were as follows: histological subtype of squamous cell carcinoma (SCC), adenocarcinoma (ADC), or adenosquamous carcinoma (ASC); stage I-IIA according to the 2009 FIGO staging system; underwent radical surgery, followed by postoperative RT alone or CCRT; and availability of complete clinicopathological and treatment data. Patients with any high-risk factors, including metastasis of pelvic lymph node(s), positive resection margin(s), parametrial invasion, and others who did not fulfill the inclusion criteria were excluded.

### Clinicopathological variables and risk stratification

Clinicopathological data, including age at diagnosis, FIGO stage, histological type, differentiation, tumor size, depth of stromal invasion, and LVSI, were retrospectively collected for analyses.

In terms of risk stratification, patients with any intermediate-risk factors, such as large tumor size (≥ 4 cm), deep stromal invasion (≥ 1/2), and LVSI, were categorized as the intermediate-risk group, while the remaining patients were classified as the low-risk group.

### Therapy

The primary therapy for all patients was radical hysterectomy with resection of the bilateral pelvic lymph nodes ± para-aortic lymphadenectomy, which was performed by experienced gynecological oncologists.

Adjuvant RT and CCRT was initiated within 4–6 weeks postoperatively with or without intracavitary brachytherapy. External beam radiation therapy was administered to the entire pelvic cavity, with or without para-aortic lymph node region, with a dose ranging from 45 Gy to 50.4 Gy in 25–28 fractions using intensity modulated radiation therapy. Intracavitary brachytherapy was administered to most patients at a dose of 10–20 Gy in 2–4 fractions.

Concurrent cisplatin-based chemotherapy was added to some patients at the discretion of the oncologists. The most common regimen was cisplatin (40 mg/m^2^) once per week for 4–6 cycles. Other regimens, including cisplatin plus paclitaxel, or cisplatin plus 5-fluorouracil, were administered to patients every 3 weeks for 2–3 cycles by intravenous infusion.

### Follow-up and endpoints

Post-treatment follow-up was performed every 3 months in the first 2 years, every 6 months in the next 3 years, and then once per year thereafter by both experienced gynecological and radiation oncologists. Regular follow-up data, including physical examination, routine laboratory investigations, hepatorenal function, serum tumor markers (SCC antigen, carbohydrate antigen 125), cervical thinprep cytology test (TCT), and imaging examinations (ultrasonography, computed tomography [CT], magnetic resonance imaging [MRI]), were obtained from outpatient and inpatient medical records, and by contacting the physician and patient family members. Biopsy for pathology and positron emission tomography-CT were performed for patients with a high suspicion of recurrence or metastasis.

The primary endpoints of treatment were overall survival (OS), referring to the interval from radical surgery to any cause of death or the most recent follow-up, and disease-free survival (DFS), defined as the interval from radical surgery to the first recurrence or most recent contact.

Treatment-related toxicity was evaluated according to the Common Toxicity Criteria for Adverse Events (CTCAE) version 5.0, which mainly involved the hematological, gastrointestinal, and urogenital systems.

### Statistical analysis

Chi-squared or Fisher’s exact tests were used to calculate frequency distributions and compare basic clinicopathological characteristics between patients who underwent RT alone versus CCRT. The Kaplan–Meier method and log-rank test were used to calculate 5-year OS and DFS rates and to generate survival curves for patients in the intermediate- and low-risk groups. Independent prognostic factors for OS and DFS were evaluated using univariate and multivariate Cox regression analyses. Furthermore, to reduce possible selection bias, 1:1 propensity score matching was performed to balance baseline variables between patients undergoing RT and CCRT in the intermediate-risk group. All statistical analyses were performed using SPSS version 23.0 (IBM Corporation, Armonk, NY, USA). Differences with a two-sided *p* < 0.05 were considered to be statistically significant.

## Results

### Clinicopathological characteristics

**Fig. 1:**
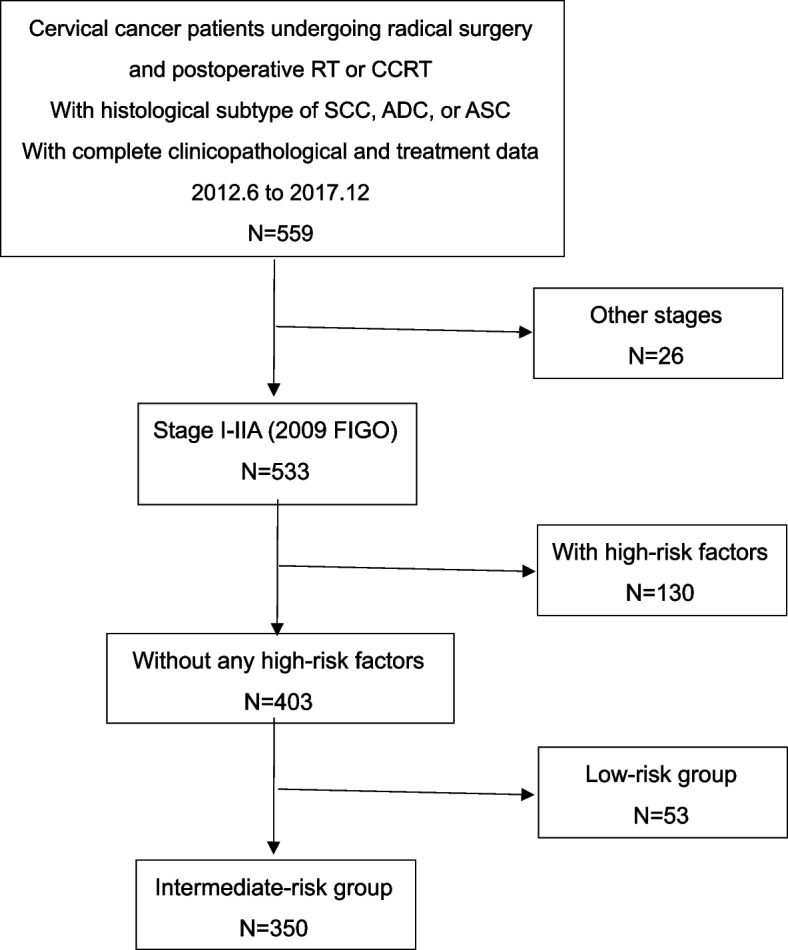
The flow-process diagram of patients’ selection

The flow-process diagram of patients’ selection is shown in Fig. 1. A total of 403 patients were enrolled in the present study and divided into 2 groups according to postoperative adjuvant treatment: RT alone (*n* = 105 [26.1%]); and CCRT (*n* = 298 [73.9%]). Clinicopathological characteristics of all patients are summarized in Table 1. Patients undergoing adjuvant CCRT tended to be younger than those undergoing RT alone (*p* = 0.035). According to FIGO stage, 8 (2.0%) patients were IA, 352 (87.3%) were IB and 43 (10.7%) were IIA. Regarding histology, most patients had SCC (80.9%), followed by ADC (13.9%), and ASC (5.2%), which were similar between the adjuvant RT and CCRT groups.

**Table 1:** Clinicopathological characteristics of all the patients

Characteristics	Total (*n* = 403)	RT (*n* = 105)	CCRT (*n* = 298)	*p*-value
**Age (yr)**				
< 45	193 (47.9%)	41 (39.0%)	152 (51.0%)	0.035
≥ 45	210 (52.1%)	64 (61.0%)	146 (49.0%)	
**FIGO stage (2009)**				
IA	8 (2.0%)	2 (1.9%)	6 (2.0%)	0.761
IB	352 (87.3%)	94 (89.5%)	258 (86.6%)	
IIA	43 (10.7%)	9 (8.6%)	34 (11.4%)	
**Histology**				
SCC	326 (80.9%)	84 (80.0%)	242 (81.2%)	0.952
ADC	56 (13.9%)	15 (14.3%)	41 (13.8%)	
ASC	21 (5.2%)	6 (5.7%)	15 (5.0%)	
**Risk stratification**				
Intermediate-risk group	350 (86.8%)	84 (80.0%)	266 (89.3%)	0.016
Number of intermediate-risk factor				
1	190 (54.3%)	58 (69.0%)	132 (49.6%)	0.004
2	124 (35.4%)	23 (27.4%)	101 (38.0%)	
3	36 (10.3%)	3 (3.6%)	33 (12.4%)	
Low-risk group	53 (13.2%)	21 (20.0%)	32 (10.7%)	
**Differentiation**				
Well differentiated	69 (17.1%)	20 (19.0%)	49 (16.4%)	0.678
Moderately differentiated	171 (42.4%)	41 (39.0%)	130 (43.6%)	
Poorly/undifferentiated	122 (30.3%)	29 (27.6%)	93 (31.2%)	
Unknown	41 (10.2%)	15 (14.3%)	26 (8.7%)	
**Tumor size (cm)**				
< 4	266 (66.0%)	79 (75.2%)	187 (62.8%)	0.020
≥ 4	137 (34.0%)	26 (24.8%)	111 (37.2%)	
**Depth of stromal invasion**				
< 1/2	163 (40.4%)	51 (48.6%)	112 (37.6%)	0.049
≥ 1/2	240 (59.6%)	54 (51.4%)	186 (62.4%)	
**LVSI**				
Negative	234 (58.1%)	72 (68.6%)	162 (54.4%)	0.011
Positive	169 (41.9%)	33 (31.4%)	136 (45.6%)	
**Neoadjuvant chemotherapy**				
No	319 (79.2%)	84 (80.0%)	235 (78.9%)	0.805
Yes	84 (20.8%)	21 (20.0%)	63 (21.1%)	

*RT* radiotherapy, *CCRT* concurrent radiochemotherapy, *FIGO* International Federation of Gynecology and Obstetrics, *SCC* squamous cell carcinoma, *ADC* adenocarcinoma, *ASC* adenosquamous carcinoma, *LVSI* lymphovascular space involvement

For risk stratification, patients were also divided into 2 groups: intermediate-risk (*n* = 350 [86.8%]); and low-risk (*n* = 53 [13.2%]). Patients in the intermediate-risk group and those with multiple intermediate-risk factors were more likely to undergo postoperative CCRT than RT alone (*p* = 0.016 and *p* = 0.004).

### Survival outcomes

The overall median follow-up was 51.7 months (range, 0.6–102.9 months). For patients in the intermediate-risk group, the median follow-up was 50.4 months (range, 0.6–102.9 months) and 64.6 months (range, 3.3–98.7 months) for those in the low-risk group.

**Fig. 2:**
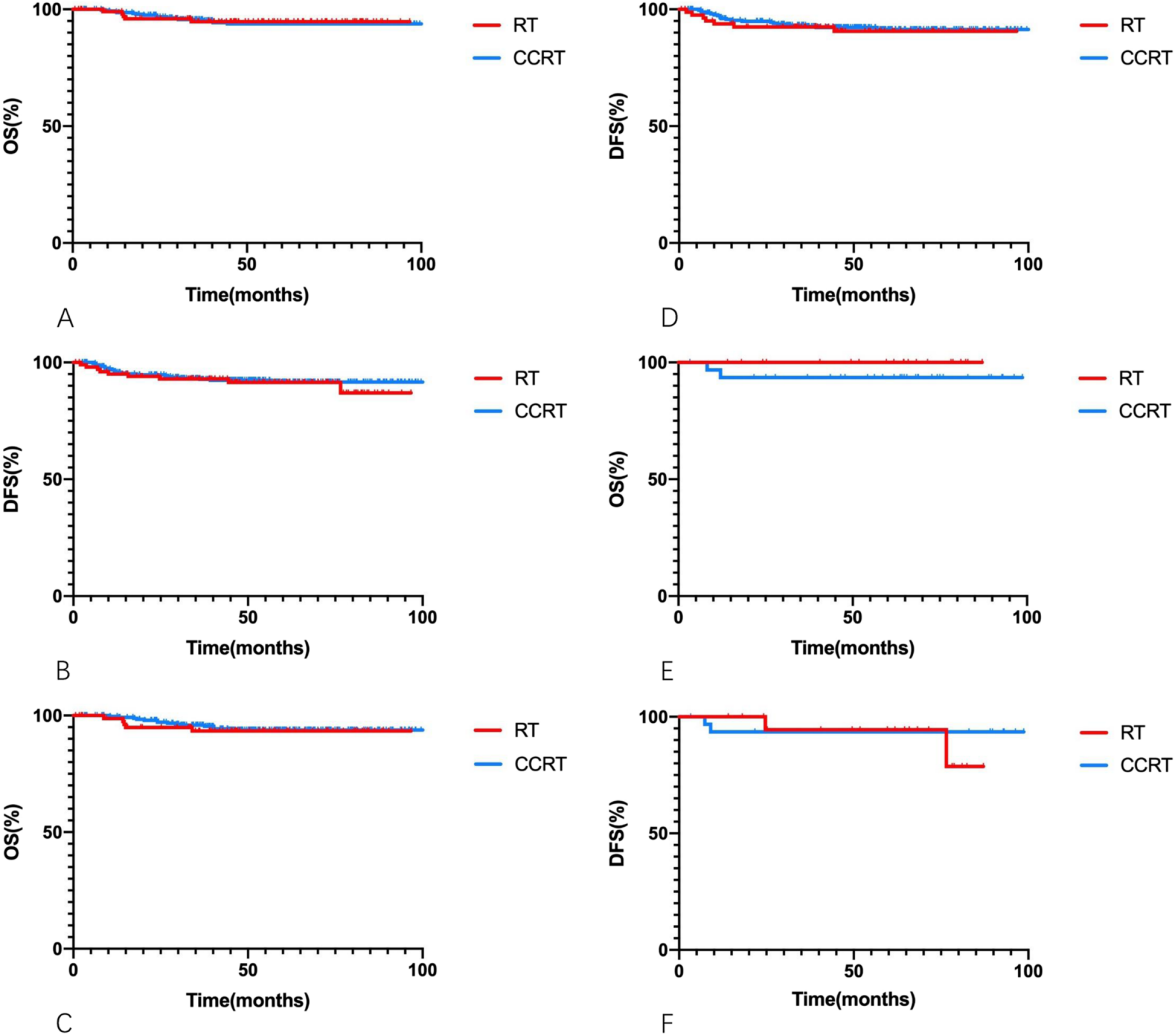
Kaplan–Meier curves of overall survivial (OS) and disease-free survival (DFS) rates for all the patients (**A**, **B**), patients in the intermediate-risk group (**C**, **D**), and patients in the low-risk group (**E**, **F**) underwent postoperative RT alone or CCRT

For all patients who underwent RT alone or CCRT, the 5-year OS rates were 94.7% and 93.8% (*p* = 0.861), the 5-year DFS rates were 91.5% and 91.7%, respectively (*p* = 0.672) (Table 2). Kaplan–Meier curves are presented in Fig. 2.

**Table 2:** 5-year OS and DFS rate of patients underwent postoperative RT alone or CCRT

5-year rate	Intermediate-risk group			Low-risk group			Intermediate and low-risk group		
	RT	CCRT	*p*-value	RT	CCRT	*p*-value	RT	CCRT	*p*-value
**OS**	93.4%	93.8%	0.741	100.0%	93.5%	0.241	94.7%	93.8%	0.861
**DFS**	90.6%	91.4%	0.733	94.4%	93.5%	0.736	91.5%	91.7%	0.672

*OS* overall survivial, *DFS* disease-free survival, *RT* radiotherapy, *CCRT* concurrent radiochemotherapy

#### Intermediate-risk group

For patients in the intermediate-risk group who underwent postoperative RT alone or CCRT, the 5-year OS rates were 93.4% and 93.8% (*p* = 0.741), and the 5-year DFS rates were 90.6% and 91.4% (*p* = 0.733) (Table 2). Kaplan–Meier curves are presented in Fig. 2.

The clinicopathological characteristics of patients in the intermediate-risk group are summarized in Table 3. Propensity score matching (1:1) was used to balance basic characteristics (including age at diagnosis, number of intermediate-risk factors, tumor size, and LVSI) between patients receiving adjuvant RT alone versus CCRT in the intermediate-risk group, which resulted in 83 patients in the RT group matched with 83 in the CCRT group (Table 3). For patients undergoing postoperative RT alone versus CCRT after matching, the 5-year OS rates were 93.3% and 96.0% (*p* = 0.409) and the 5-year DFS rates were 90.5% and 93.8%, respectively (*p* = 0.287).

**Table 3:** Clinicopathological characteristics of patients in the intermediate-risk group before and after 1:1 propensity score matching

Characteristics	Before 1:1 propensity score matching			After 1:1 propensity score matching		
	**RT (*n* = 84)**	**CCRT (*n* = 266)**	***p*-value**	**RT (*n* = 83)**	**CCRT (*n* = 83)**	***p*-value**
**Age (yr)**						
< 45	32 (38.1%)	137 (51.5%)	0.032	32 (38.6%)	43 (51.8%)	0.086
≥ 45	52 (61.9%)	129 (48.5%)		51 (61.4%)	40 (48.2%)	
**FIGO stage (2009)**						
IA	2 (2.4%)	5 (1.9%)	0.376	2 (2.4%)	4 (4.8%)	0.062
IB	77 (91.7%)	231 (86.8%)		76 (91.6%)	65 (78.3%)	
IIA	5 (6.0%)	30 (11.3%)		5 (6.0%)	14 (16.9%)	
**Histology**						
SCC	67 (79.8%)	225 (84.6%)	0.394	66 (79.5%)	69 (83.1%)	0.404
ADC	11 (13.1%)	30 (11.3%)		11 (13.3%)	12(14.5%)	
ASC	6 (7.1%)	11 (4.1%)		6 (7.2%)	2 (2.4%)	
**Number of intermediate risk factor**						
1	58 (69.0%)	132 (49.6%)	0.004	58 (69.9%)	69 (83.1%)	0.099
2	23 (27.4%)	101 (38.0%)		22 (26.5%)	11 (13.3%)	
3	3 (3.6%)	33 (12.4%)		3 (3.6%)	3 (3.6%)	
**Differentiation**						
Well differentiated	19 (22.6%)	42 (15.8%)	0.155	19 (22.9%)	14 (16.9%)	0.193
Moderately differentiated	35 (41.7%)	120 (45.1%)		34 (41.0%)	32 (38.6%)	
Poorly/undifferentiated	18 (21.4%)	82 (30.8%)		18 (21.7%)	29 (34.9%)	
Unknown	12 (14.3%)	22 (8.3%)		12 (14.5%)	8 (9.6%)	
**Tumor size (cm)**						
< 4	58 (69.0%)	155 (58.3%)	0.078	58 (69.9%)	58 (69.9%)	1.000
≥ 4	26 (31.0%)	111 (41.7%)		25 (30.1%)	25 (30.1%)	
**Depth of stromal invasion**						
< 1/2	30 (35.7%)	80 (30.1%)	0.332	29 (34.9%)	39 (47.0%)	0.114
≥ 1/2	54 (64.3%)	186 (69.9%)		54 (65.1%)	44 (53.0%)	
**LVSI**						
Negative	51 (60.7%)	130 (48.9%)	0.058	51 (61.4%)	52 (62.7%)	0.873
Positive	33 (39.3%)	136 (51.1%)		32 (38.6%)	31 (37.3%)	
**Neoadjuvant chemotherapy**						
No	66 (78.6%)	208 (78.2%)	0.942	65 (78.3%)	66 (79.5%)	0.849
Yes	18 (21.4%)	58 (21.8%)		18 (21.7%)	17 (20.5%)	

*RT* radiotherapy, *CCRT* concurrent radiochemotherapy, *FIGO* International Federation of Gynecology and Obstetrics, *SCC* squamous cell carcinoma, *ADC* adenocarcinoma, *ASC* adenosquamous carcinoma, *LVSI* lymphovascular space involvement

**Fig. 3:**
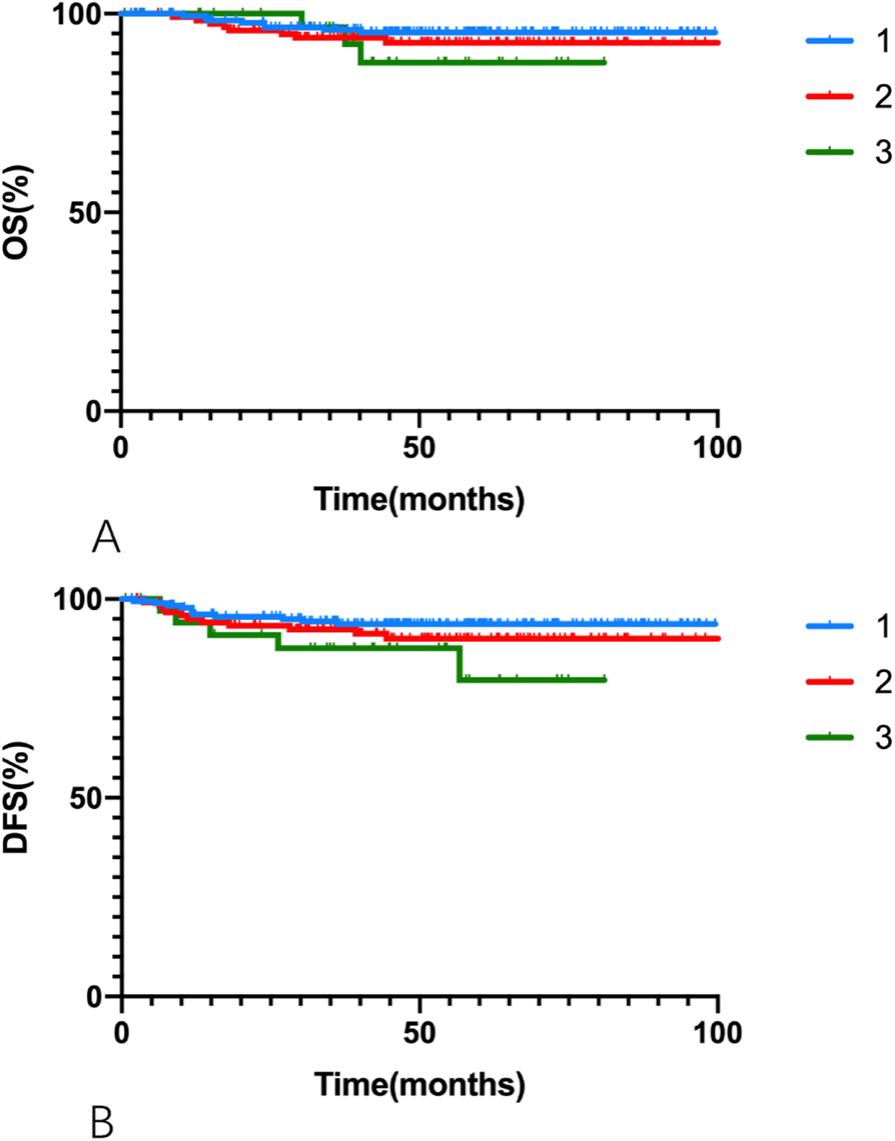
Kaplan–Meier curves of (**A**) overall survivial (OS) and (**B**) disease-free survival (DFS) rates for patients in the intermediate-risk group stratified by number of intermediate-risk factors

Of the 350 patients in the intermediate-risk group, 190 (54.3%) had a single intermediate-risk factor, 124 (35.4%) had 2, and 36 (10.3%) had 3. For patients with 1, 2, and 3 intermediate-risk factors, the 5-year OS rates were 95.3%, 92.7%, and 87.7%, respectively (*p* = 0.434). The 5-year DFS rates were 93.7%, 90.0%, and 79.6%, respectively (*p* = 0.166). Kaplan–Meier curves are presented in Fig. 3.

**Fig. 4:**
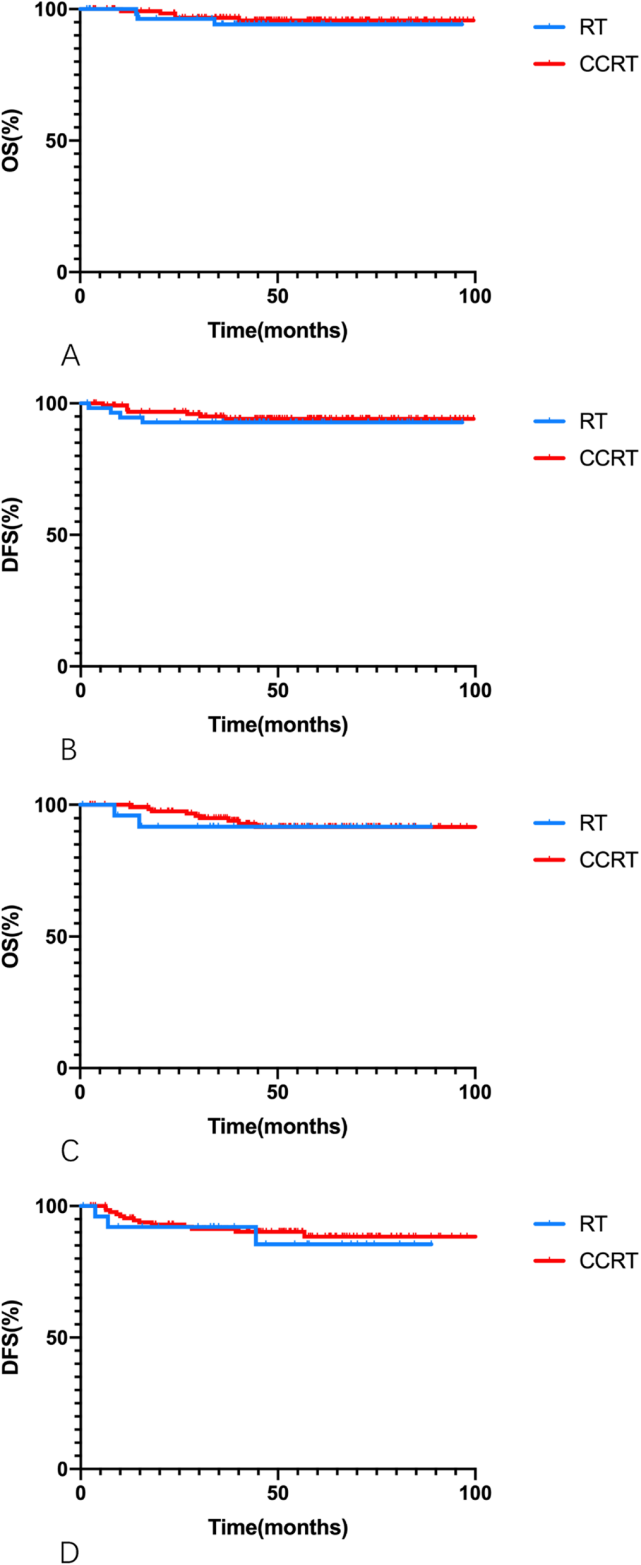
Kaplan–Meier curves of overall survivial (OS) and disease-free survival (DFS) rates for patients with single intermediate-risk factor (**A**, **B**), and multiple intermediate-risk factors (**C**, **D**) underwent postoperative RT alone or CCRT

For patients with only 1 intermediate-risk factor who underwent RT alone versus CCRT, the 5-year OS rates were 94.2% and 95.7% (*p* = 0.636), and the 5-year DFS rates were 92.8% and 94.1%, respectively (*p* = 0.637). For patients with ≥ 2 intermediate-risk factors undergoing RT alone versus CCRT, the 5-year OS rates were 91.8% and 91.7% (*p* = 0.761), and the 5-year DFS rates were 85.4% and 88.4%, respectively (*p* = 0.717). Kaplan–Meier curves are presented in Fig. 4.

#### Low-risk group

Among patients in the low-risk group undergoing RT alone versus CCRT, the 5-year OS and DFS rates were 100.0% and 93.5% (*p* = 0.241), versus 94.4% and 93.5% (*p* = 0.736), respectively (Table 2). Kaplan–Meier curves are presented in Fig. 2.

### Prognostic factors

In univariate analysis (Table 4), postoperative adjuvant therapy (i.e., CCRT or RT) was not associated with OS (*p* = 0.861) or DFS (*p* = 0.673). In multivariate analysis, postoperative adjuvant therapy was not an independent factor for either OS (*p* = 0.858) or DFS (*p* = 0.366) (Table 5).

**Table 4:** Univariate Cox regression analysis of prognostic factors for all the patients

Variables	OS		DFS	
	**HR (95% CI)**	***p*-value**	**HR (95% CI)**	***p*-value**
Age (< 45 vs ≥ 45)	0.581 (0.241–1.401)	0.226	0.678 (0.332–1.383)	0.285
FIGO stage (2009) (I vs IIA)	0.041 (0.000–14.338)	0.286	0.535 (0.128–2.241)	0.392
Histology (SCC vs AC)	1.596 (0.619–4.115)	0.333	1.665 (0.767–3.616)	0.198
Risk stratification (Intermediate vs Low)	0.671 (0.156–2.883)	0.592	0.918 (0.321–2.625)	0.873
Tumor size(cm) (< 4 vs ≥ 4)	2.691 (1.134–6.388)	0.025	2.160 (1.068–4.369)	0.032
Depth of stromal invasion(< 1/2 vs ≥ 1/2)	1.129 (0.468–2.725)	0.787	1.107 (0.537–2.280)	0.784
LVSI (Negative vs Positive)	0.877 (0.363–2.116)	0.770	1.060 (0.518–2.166)	0.874
Neoadjuvant chemotherapy (No vs Yes)	0.805 (0.271–2.393)	0.696	0.838 (0.344–2.044)	0.698
Postoperative treatment (RT vs CCRT)	1.094 (0.401–2.987)	0.861	0.846 (0.389–1.838)	0.673

*OS* overall survivial, *DFS* disease-free survival, *HR* hazard ratio, *CI* confidence interval, *FIGO* International Federation of Gynecology and Obstetrics, *SCC* squamous cell carcinoma, *AC* adenocarcinoma and adenosquamous carcinoma, *LVSI* lymphovascular space involvement, *RT* radiotherapy, *CCRT* concurrent radiochemotherapy

**Table 5:** Multivariate Cox regression analysis of prognostic factors for all the patients

Variables	OS		DFS	
	**HR (95% CI)**	***p*-value**	**HR (95% CI)**	***p*-value**
Age (< 45 vs ≥ 45)	0.521 (0.212–1.280)	0.155	0.634 (0.305–1.315)	0.221
Histology (SCC vs AC)	1.786 (0.662–4.818)	0.252	1.857 (0.825–4.179)	0.135
Risk stratification (Intermediate vs Low)	0.995 (0.159–6.229)	0.995	1.675 (0.408–6.881)	0.474
Tumor size(cm) (< 4 vs ≥ 4)	3.489 (1.307–9.313)	0.013	3.031 (1.356–6.774)	0.007
Depth of stromal invasion(< 1/2 vs ≥ 1/2)	1.012 (0.361–2.834)	0.982	1.252 (0.520–3.014)	0.616
LVSI (Negative vs Positive)	0.938 (0.351–2.510)	0.899	1.360 (0.602–3.074)	0.459
Neoadjuvant chemotherapy (No vs Yes)	0.523 (0.160–1.709)	0.283	0.670 (0.257–1.744)	0.412
Postoperative treatment (RT vs CCRT)	0.910 (0.322–2.569)	0.858	0.688 (0.307–1.546)	0.366

*OS* overall survivial, *DFS* disease-free survival, *HR* hazard ratio, *CI* confidence interval, *SCC* squamous cell carcinoma, *AC* adenocarcinoma and adenosquamous carcinoma, *LVSI* lymphovascular space involvement, *RT* radiotherapy, *CCRT* concurrent radiochemotherapy

### Treatment-related toxicity

During follow-up, 161 (40.0%) patients developed grade ≥ 3 treatment-related toxicities, including 21 (20.0%) patients in the RT group and 140 (47.0%) in the CCRT group (*p* < 0.001) (Table 6). It is noteworthy that patients undergoing adjuvant CCRT appeared to develop a higher proportion of grade ≥ 3 acute hematological toxicities than those undergoing RT alone (44.0% and 11.4%, respectively; *p* < 0.001). Moreover, there was no significant difference in grade ≥ 3 chronic toxicities of the urogenital and gastrointestinal systems between the 2 groups (*p* = 0.845 and *p* = 1.000).

**Table 6:** Toxicities related to treatment of all the patients

Toxicities	Total (*n* = 403)	RT (*n* = 105)	CCRT (*n* = 298)	*p*-value
**≥ Grade 3**	161 (40.0%)	21 (20.0%)	140 (47.0%)	< 0.001
Acute Hematologic	143 (35.5%)	12 (11.4%)	131 (44.0%)	< 0.001
Chronic Urogenital	29 (7.2%)	8 (7.6%)	21 (7.0%)	0.845
Chronic Gastrointestinal	8 (2.0%)	2 (1.9%)	6 (2.0%)	1.000

*RT* radiotherapy, *CCRT* concurrent radiochemotherapy

### SCC versus ADC/ASC

#### Patients with SCC

The 5-year OS rates for patients with SCC in the intermediate-risk group who underwent RT alone versus CCRT were 91.6% and 95.3% (*p* = 0.206), and the 5-year DFS rates were 88.1% and 93.3%, respectively (*p* = 0.146). The 5-year OS rates for patients with SCC in the low-risk group undergoing RT alone versus CCRT were 100.0% and 93.8% (*p* = 0.303), and the 5-year DFS rates were 92.9% and 93.8%, respectively (*p* = 0.983).

#### Patients with ADC/ASC

The 5-year OS rates for patients with ADC/ASC in the intermediate-risk group who underwent RT alone versus CCRT were 100.0% and 86.0% (*p* = 0.143), and the 5-year DFS rates were 100.0% and 81.0%, respectively (*p* = 0.075). The 5-year OS rates for patients with ADC/ASC in the low-risk group undergoing RT alone versus CCRT were 100.0% and 93.3% (*p* = 0.606), the 5-year DFS rates were 100.0% and 93.3%, respectively (*p* = 0.541).

## Discussion

This retrospective study analyzed and compared clinicopathological characteristics, survival outcomes, and treatment-related toxicities between patients without high-risk factors who underwent RT alone versus CCRT after radical surgery.

Several studies have compared clinicopathological features between patients who underwent postoperative RT alone versus CCRT. Mahmoud et al. [10] found that, for early-stage cervical cancer patients with intermediate-risk factors, younger patients with higher FIGO stage tended to undergo adjuvant chemoradiotherapy. However, another study demonstrated no significant difference in age and FIGO stage between the RT and CCRT groups. Furthermore, there was a greater tendency for patients with multiple intermediate-risk factors to undergo postoperative CCRT than RT alone [11]. According to our experience, compared with patients in the RT group, those in the CCRT group were younger and exhibited a higher proportion of multiple intermediate-risk factors.

Based on clinical trials and long-term follow up, adjuvant CCRT is recommended for early-stage cervical cancer patients with high-risk factors following radical surgery [2, 3, 13, 14]. However, for early-stage patients without high-risk factors, no consensus has been reached regarding whether postoperative CCRT is superior to RT alone.

In recent years, however, a series of studies have come to different conclusions. Song et al. [8] enrolled 110 patients with stage IB-IIA cervical cancer with ≥ 2 intermediate-risk factors who underwent RT (*n* = 56) or CCRT (*n* = 54) following radical surgery. Results revealed that, for patients who underwent RT or CCRT between 2000 and 2010, the 5-year OS rates were 83.0% and 92.9% (*p* = 0.030), and the 5-year relapse-free survival rates were 85.6% and 93.8% (*p* = 0.003). Additionally, Kim et al. [15] retrospectively reviewed the medical records of 79 patients (postoperative chemoradiation [*n* = 55] versus RT [*n* = 24]), and Mabuchi et al. [13] also enrolled 57 stage IA2-IIB patients with intermediate-risk factors. Both studies demonstrated that for patients with intermediate-risk factors, postoperative CCRT was more effective than RT alone in improving OS and progression-free survival (PFS). However, the insufficient sample sizes and patient heterogeneity were limitations that cannot be ignored.

In contrast, more recent studies with long-term follow up and large sample sizes have reported that postoperative CCRT was not superior to RT alone for early-stage patients with intermediate-risk factors [10, 11]. Kim et al. [11] retrospectively reviewed 316 stage IB-IIA cervical cancer patients with intermediate-risk factors who underwent CCRT or RT after radical surgery, with a median follow-up of 70 months. Results revealed that for patients who underwent RT alone and CCRT, the 5-year recurrence-free survival (RFS) was 90.8% and 88.9% (*p* = 0.631), and 5-year OS was 95.9% and 91.0% (*p* = 0.287). Moreover, a meta-analysis in 2016, including 1073 patients from 11 studies with 582 patients in the CCRT group and 491 in the RT alone group, demonstrated that for those with intermediate-risk factors, there was no benefit for either OS or PFS when concurrent chemotherapy was added to adjuvant RT [12]. Based on the large sample size and long follow-up period in our institution, our research demonstrated that CCRT was not superior to RT alone in improving 5-year OS and DFS rates for patients with cervical cancer without high-risk factors. Nevertheless, prospective, randomized, and multicenter studies with sufficient sample sizes are warranted.

In terms of the number of intermediate-risk factors, Okazawa et al. [16] found that for IB1-IIB patients with multiple intermediate-risk factors, postoperative CCRT was more effective than RT. For patients with a single intermediate-risk factor, there was no survival benefit with CCRT compared with RT. However, the study enrolled patients with stage IIB, which may increase the heterogeneity. Our study further demonstrated that postoperative CCRT was not more effective than RT in patients stratified according to the number of intermediate-risk factors, which was consistent with the study by Kim et al. [11].

For patients who underwent definitive RT or CCRT, the prognosis of patients with ADC/ASC was worse than those with SCC [17–19]. Seki et al. [20] found that, for stage IB-IIB patients with ADC/ASC after radical surgery, compared with patients who underwent adjuvant chemotherapy, PFS was shorter for those underwent adjuvant RT/CCRT, which suggested that the radiosensitivity of ADC/ASC may be lower than that for SCC [21]. Moreover, Zhou et al. [21] demonstrated that for early-stage cervical cancer patients with positive nodes, the survival benefit of postoperative CCRT over RT was only observed in those with SCC and not ADC. However, our study showed that, for patients with early-stage cervical cancer without any high-risk factors, treatment outcomes between RT versus CCRT were similar regardless of histological subtype (i.e., SCC, ADC/ASC).

The concurrent chemotherapy regimen in most previous studies was cisplatin-based therapy [11, 12]. Kim et al. [11] found that, compared with patients undergoing RT alone, patients who underwent cisplatin-based postoperative CCRT experienced a higher proportion of grade ≥ 3 hematological, gastrointestinal, and genitourinary toxicity. Based on a retrospective study by Song et al. [8] and a randomized phase III trial (NCT01418859) by Sun et al. [22], a concurrent chemotherapy regimen of paclitaxel and carboplatin for most patients, and topotecan and cisplatin, respectively, the investigators demonstrated that compared with RT group, patients who underwent CCRT developed more frequent hematological toxicity. However, there was no significant difference in grade 3–4 non-hematological toxicities between the CCRT and RT groups. In our study, most patients underwent weekly cisplatin for concurrent chemotherapy, and results indicated that those who underwent CCRT developed a higher proportion of grade ≥ 3 acute hematological toxicities than those in the RT group. However, there was no significant difference between the 2 groups in terms of chronic gastrointestinal and urogenital toxicities. Future studies should focus on developing an effective and well-tolerated postoperative treatment regimen. The therapeutic effects and complications of different concurrent chemotherapy regimens clearly merit further study.

Our study had some limitations. Firstly, this study is retrospective in nature, and some patients who do not meet the Sedlis Criteria also underwent postoperative radiotherapy. We currently lack sufficient evidence to endorse this risk classification system, particularly when compared to the Sedlis criteria. Secondly, some of the patients underwent neoadjuvant chemotherapy, which may have introduced heterogeneity to the treatment outcomes. As such, prospective, multi-institution, and randomized clinical trials are warranted.

## Conclusion

In this study, we found that for patients with early-stage cervical cancer without any high-risk factors, there was no significant difference in 5-year OS or DFS rates between postoperative adjuvant CCRT and RT. Patients who underwent postoperative CCRT were more likely to develop grade ≥ 3 acute hematological toxicities versus those who underwent RT alone.

## Data Availability

The datasets used and analyzed during the current study are available from the corresponding author on reasonable request.

## Abbreviations

CCRT: Concurrent radiochemotherapy
RT: Radiotherapy
OS: Overall survival
DFS: Disease-free survival
NCCN: National Comprehensive Cancer Network
FIGO: International Federation of Gynecology and Obstetrics
DSI: Deep stromal invasion
LVSI: Lymphovascular space involvement
SCC: Squamous cell carcinoma
ADC: Adenocarcinoma
ASC: Adenosquamous carcinoma
EBRT: External beam radiation therapy
TCT: Thinprep cytology test
CT: Computed tomography
MRI: Magnetic resonance imaging
PET: Positron emission tomography
CTCAE: Common Toxicity Criteria for Adverse Events
HR: Hazard ratio
CI: Confidence interval
PFS: Progression-free survival
RFS: Recurrence-free survival
